# Concurrence of primary pulmonary malignant melanoma with invasive pulmonary adenocarcinoma: a case report

**DOI:** 10.1186/s13019-020-01091-5

**Published:** 2020-03-26

**Authors:** Luhuan Yang, Yunhong Lei, Rong Zhang, Yufei Liu, Wenli Dai, Fei Tian, Jinglan Liu

**Affiliations:** 1 Department of Emergency and Critical Care Medicine, The First College of Clinical Medical Science, Three Gorges University, Yichang Central People’s Hospital, Yiling Road 183, Yichang City, 443003 Hubei Province China; 2 Department of Pathology, The First College of Clinical Medical Science, Three Gorges University, Yichang Central People’s Hospital, Yichang City, 443003 Hubei Province China; 3 Department of Nuclear Medicine, The First College of Clinical Medical Science, Three Gorges University, Yichang Central People’s Hospital, Yichang City, 443003 Hubei Province China

**Keywords:** Malignant melanoma, Adenocarcinoma, Concurrence, Primary lung tumor

## Abstract

**Background:**

Primary pulmonary malignant melanoma (PPMM) is an extreme rarity in clinic practice, accounting for only 0.01% of all primary pulmonary tumors. And its diagnosis should meet clinical and pathological diagnosis criteria in addition to excluding the possibility of metastatic melanoma. The mainstay of treatment is surgery. The concurrence of primary pulmonary malignant melanoma and invasive pulmonary adenocarcinoma has not been reported before.

**Case presentation:**

Herein we report the case of a 39-year-old woman who was asymptomatic and accidently found to have the concurrence of PPMM with invasive pulmonary adenocarcinoma. Before considering the diagnosis of primary pulmonary malignant melanoma, a systemic positron emission tomography-computed tomography (PET-CT) was done to excluding primary tumor metastasis from other sites. The pathological biopsy proved that two lesions in the right middle lobe were invasive pulmonary adenocarcinomas and the mass in the right lower lobe was malignant melanoma. She underwent right middle and lower lobectomy of the lung with mediastinal and hilar lymph dissection. She refused adjuvant chemotherapy, genetic molecular testing or immunotherapy. Fifteen months later she had brain metastasis. Then she received brain radiotherapy and underwent follow-up at the outpatient clinic regularly.

**Conclusions:**

We experienced a case of concurrent PPMM and invasive pulmonary adenocarcinoma. The patient reported here is the first case of primary pulmonary malignant melanoma combined with invasive pulmonary adenocarcinoma. This patient remained disease-free 15 months after lung surgery.

## Background

Malignant melanoma (MM) is a malignant neoplasm of the melanocytes, usually arising from the skin. But it may also occur in other mucosal sites and organs [1–3]. Primary pulmonary malignant melanoma (PPMM) is extremely rare as only 51 cases have been reported since 1916 [2, 4]. The concurrence of PPMM and pulmonary adenocarcinoma has hardly been reported so far. Herein, we present a special case who had concurrent PPMM and invasive pulmonary adenocarcinoma. And then the PPMM-related literature was reviewed.

## Case presentation

**Fig. 1:**
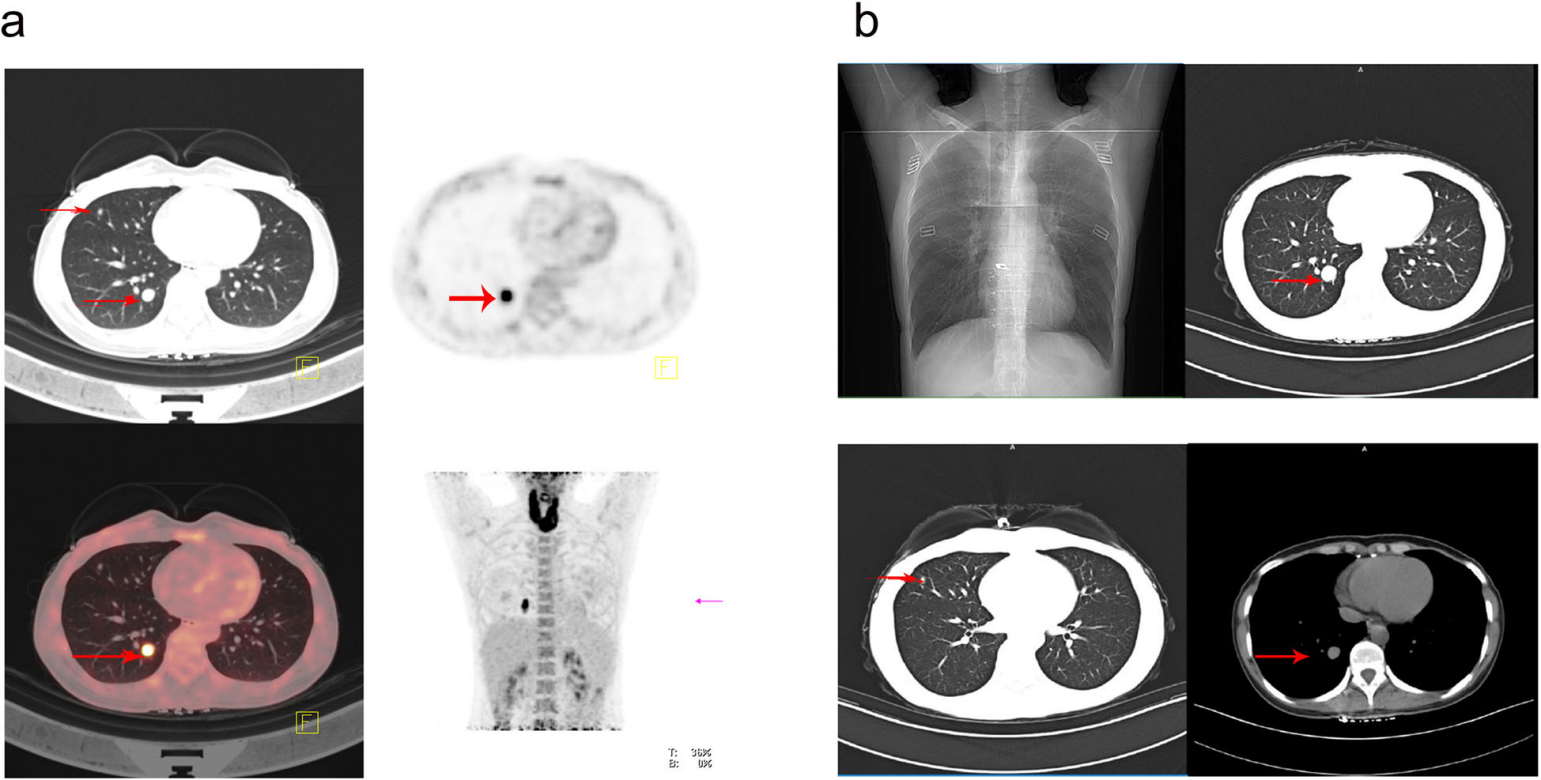
**a** PET-CT revealed multiple nodules in the right middle lobe of lung and a nodule with abnormal metabolism increase in the right lower lobe; **b** Chest CT showed the tumor’s size in the right lower lobe was increased

A 39-year-old woman with pulmonary space occupying lesions for 6 months was admitted to our hospital on January 02, 2018. She was firstly found to have pulmonary multiple nodules (maximum diameter 1.3 cm, in the right lower lobe) on the chest computed tomography (CT) 6 months before. And 3 days before admission, her chest CT revealed the lesion in the lower lobe grew to 1.5 cm. The positron emission tomography-computed tomography (PET-CT) showed the nodule in the right lower lobe had abnormal metabolism increase, which was different from the multiple nodules in the right middle lobe (Fig. 1a). And the PET-CT suggested no other possibility for a primary tumor lesion site.

The patient had no clinical symptoms. She denied any past history of smoking, skin or ocular lesions. A comprehensive physical examination was performed on admission. Her axillary temperature was 36.6 °C, blood pressure was 116/76 mmHg, pulse rate was 78 beats per minute, respiratory rate was 20 per minute, and oxygen saturation was 100% at room air. Her blood routine test, liver function test, electrolyte and renal function test, and lung cancer markers including Carcinoembryonic antigen (CEA), neuron-specific enolase (NSE), squamous cell carcinoma (SCC), CYFRA21-1 were all within the normal range, as were thyroid function test, coagulation function test, and pulmonary function test. The rapid test screening results for HIV, hepatitis B, and syphilis were negative. No abnormality was seen in her electrocardiogram. After excluding surgical contraindications, she underwent thoracoscopic resection of lung nodules under general anesthesia. During operation, two small nodules (around 0.3 cm in diameter) in the right middle lobe and a round, solid mass (2 cm in diameter) in the right lower lobe were removed and sent for frozen section histology. The intra-operative frozen section histology showed two small nodules in the middle lung indicated adenocarcinoma, and the nodules of the lower lung were solid short fusiform epithelioid cell nest with large hemosiderin deposition, which was considered to be non-small cell carcinoma. Then the patient converted to thoracotomy, and underwent right middle and lower lobectomy of the lung with mediastinal and hilar lymph dissection.

**Fig. 2:**
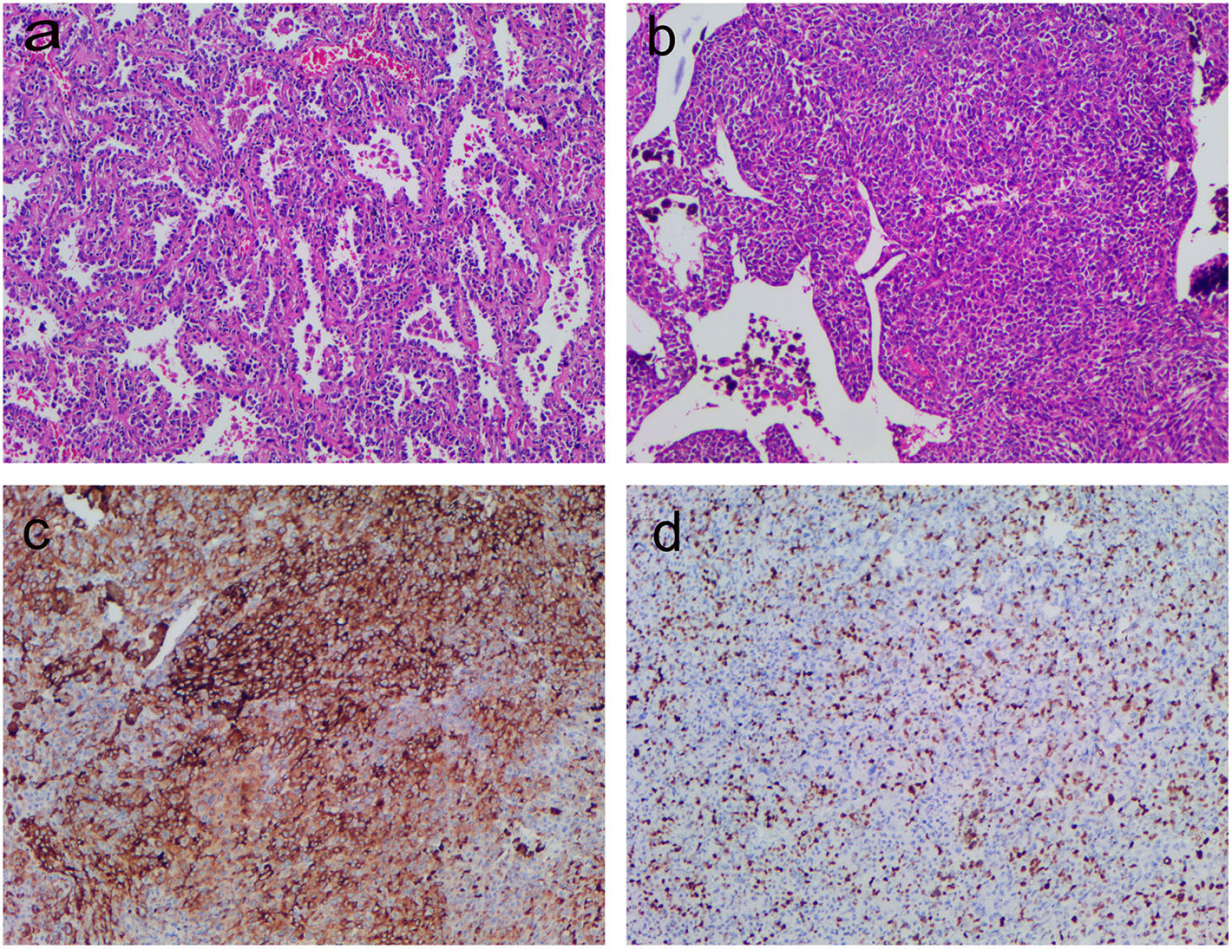
**a** Pathological section biopsy showing infiltration by lung adenocarcinoma cells with acinar and papillary mixed type (hematoxylin and eosin staining 100 x); **b** Pathological section biopsy showing infiltration by solid short fusiform epithelioid cell nests containing melanin pigmentation from the lung lesion (100x); **c** Immunohistochemical expression of HMB-45 in neoplastic cells (DAB, magnification 100x). **d** Immunohistochemical expression of Ki-67 in neoplastic cells (DAB, magnification 100x)

Four days later, the final pathological biopsy proved that two lesions in the right middle lobe of the lung were invasive pulmonary adenocarcinomas which consisted of a mixture of acinar and papillary type. The mass in the right lower lobe consisted of solid short fusiform epithelioid cell nest with large hemosiderin deposition. No tumor cells were detected in the four groups of dissected lymph nodes. Immunohistochemical (IHC) staining of the tumor in the right lower lobe was positive for human melanoma black-45 (HMB-45), Melan-A, and Ki-67 (hot spot 70%), Vimentin, S-100 protein, CgA, CDp56, whereas staining for cytokeratin (CK5/6, CK7), CD68, P63, TTF-1, P40, Napsin A, Syn, PCK [AE1/AE3] and EMA were negative (Fig. 2). Since the result of histological examination highly suggested MM and CT showed no other possibility for a primary tumor lesion site, the patient’s tumor in the right lower lobe was diagnosed as PPMM. The final diagnosis of the patient was PPMM combined with invasive pulmonary adenocarcinoma.

**Fig. 3:**
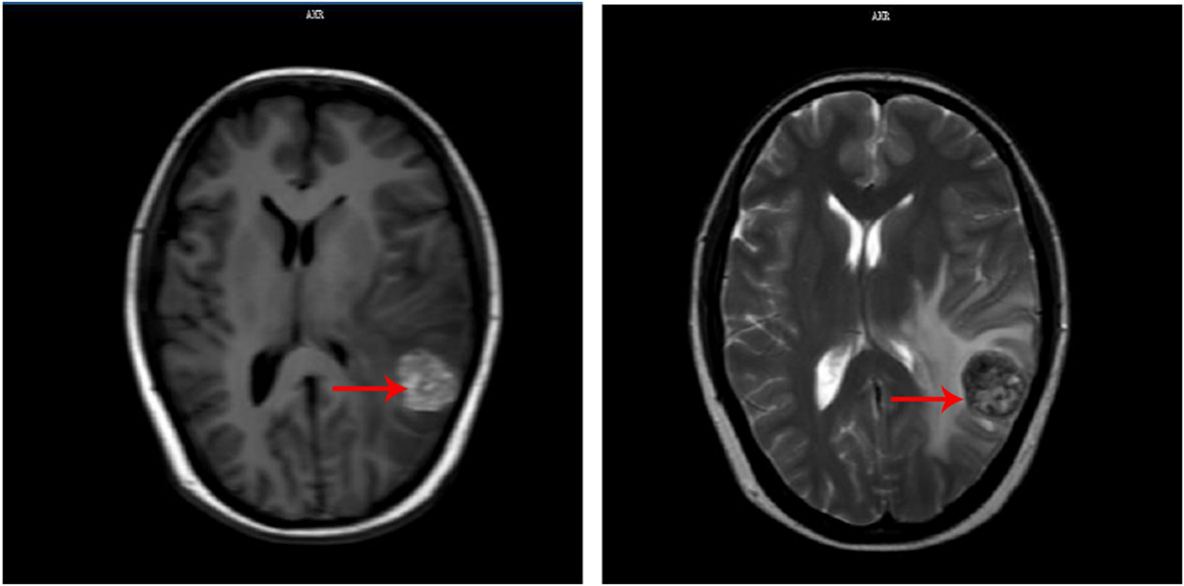
Brain-enhanced magnetic resonance showed that the left temporal lobes showed nodular high-low mixed signal, with a diameter of about 2.6 cm. Large edema was seen around the lesion, with the left lateral ventricle compressed and the midline structure shifted to the right

She refused adjuvant chemotherapy, genetic molecular testing and immunotherapy, and was discharged after recovering from the surgery. Fifteen months after the pulmonary resection, she felt dizzy and headache. Then she underwent brain magnetic resonance imaging (MRI) and chest CT. The MRI showed she had a brain metastasis (Fig. 3). She received whole-brain radiotherapy at 3 Gy each day, for a total dose of 30 Gy / 10f, together with localized radiotherapy for craniocerebral lesions with a total dose of 12.5Gy/5f. Then she was discharged and underwent follow-up at the outpatient clinic regularly.

## Discussion

There are about 160,000 cases of newly diagnosed malignant melanoma every year throughout the world, with approximately 41,000 melanoma-related deaths annually [1]. 90% of malignant melanomas arise from the skin. The neoplasm can also develop in the mucosal area, including the eye, oral mucosa, genital and perianal areas, esophagus, larynx, vagina and liver, etc. [5–7]. The pulmonary malignant melanomas are common metastatic lesions from the skin or ocular tumors [8]. PPMM is exceedingly rare, which accounts for only 0.01% of all primary pulmonary tumors [9, 10].

Kyriakopoulos et al. demonstrated the median age of PPMM was 59.1 years old (range from 29 to 90) [2, 11]. There were 21 men and 19 women in the population. Although cigarette smoking is considered as the incentive factor for lung cancers, smoking history was not found to be correlated with the development of PPMM. The common clinical manifestations include cough, hemoptysis, thoracalgia, difficult breathing, or weight loss. About 30% of patients are asymptomatic and the lesion was discovered accidentally by chest imaging examination [12].

Since PPMM is an exceptionally rare tumor, metastasic melanoma from the skin or other organs should be carefully ruled out in these conditions [13, 14]. PPMM was diagnosed according to clinical and pathological criteria [9, 11, 15]. The clinical diagnosis criteria were as follows: a central and solitary tumor in the lung; no previous excision history of a cutaneous, mucous membrane, or ocular lesion; no melanoma in the other organs at diagnosis. The pathological criteria comprise immunohistochemical staining of S-100 and HMB-45, evidence of junctional changes including “dropping off” or “nesting” arranged in fascicles, and invasion of melanoma cells into the bronchial epithelium. Our case met the above criteria for diagnosing PPMM.

Lobectomy or pneumonectomy with lymph node dissection is the main treatment for localized pulmonary malignant melanoma [11, 16, 17]. Different types of chemotherapy and immunotherapy as well as radiotherapy have been attempted for MM patients, but no postoperative adjuvant therapy has a good effect on PPMM patients [11, 18]. About 40 to 60% of skin melanomas carry BRAF gene mutations (V600E), which causes downstream signals continuous activation through the MAPK pathway [2, 13, 19]. The FDA approved a range of BRAF inhibitors, including vemurafenib and dabrafenib, as well as MEK inhibitor [20–22]. With the development of immunotherapy in last few years, the FDA approved anti- PD-1 antibodies nivolumab and pembrolizumab for treating metastatic cutaneous melanoma in 2014 [11, 23]. Targeted therapy needs to evaluate the expression of tumor PD-L1.

Our patient showed 2 lesions in the middle lobe and 1 lesion in the right lower lobe. The lesion in the right lower lobe proved to be PPMM by pathological biopsy and immunohistochemical (IHC) staining. The lesions in the right middle lobe turned out to be invasive pulmonary adenocarcinoma by pathological biopsy, with typical a mixture of acinar and papillary type. There are several cases of synchronous primary melanoma and adenocarcinoma in digestive system reported. Yet there have been no similar case reports in the lung so far. The treatment of PPMM is still controversial, and the combination of adenocarcinoma makes the treatment more difficult.

The overall outcome of primary pulmonary malignant melanoma was very dismal and 65% of patients survived up to 18 months. Only two patients survived for a long time (10 and 11 years, respectively) [2]. As with other types of tumors, a better prognosis can be obtained through early discovery. Some patients were diagnosed unexpectedly by chest imaging without any clinical symptoms [2, 24]. This was the case with our patient. She didn’t exhibit any respiratory tract symptom. The tumor was discovered accidentally by chest imaging examination. Fifteen months after the pulmonary resection, she was found to have a brain metastasis and underwent brain radiotherapy. It has been 22 months since she underwent the surgery, and she is on regular follow-up till now. Maybe the combination of adenocarcinoma accelerated the progression of her disease, which is unclear.

## Conclusion

PPMM is exceedingly rare. Currently, surgery is the primary treatment for local PPMM. In this paper we showed the first case of concurrent melanoma and adenocarcinoma in the lung. And the patient remained disease-free 15 months after lung surgery.

## Data Availability

All data generated or analyzed during this study are included in this published article.

## Abbreviations

MM: Malignant melanoma
PPMM: Primary pulmonary malignant melanoma
CT: Computed tomography
PET-CT: Positron emission tomography-computed tomography
